# Under consumers’ scrutiny - an investigation into consumers’ attitudes and concerns about nudging in the realm of health behavior

**DOI:** 10.1186/s12889-015-1691-8

**Published:** 2015-04-09

**Authors:** Astrid F Junghans, Tracy TL Cheung, Denise DT De Ridder

**Affiliations:** Department of Clinical and Health Psychology, Utrecht University Heidelberglaan 1, 3508TC Utrecht, The Netherlands

**Keywords:** Nudging, Liberal paternalism, Public policy, Health, Behavioral economics

## Abstract

**Background:**

Nudging strategies have recently attracted attention from scholars and policy makers for their potential in influencing people’s behaviors on large scales. But is the fact that nudges do not forbid any choice-options or significantly alter people’s economic incentives sufficient to conclude that nudges should be implemented? While this is discussed amongst scholars from various disciplines the voices of consumers, the target-group of nudges, remain unheard. Since understanding their knowledge about nudging and their opinions on being nudged are crucial for the evaluation of the moral appropriateness of nudging, the current study examines consumers’ knowledge of and attitudes toward nudging in general and the realm of health behavior.

**Methods:**

In this qualitative investigation in-depth semi-structured interviews with UK consumers were conducted to examine consumers’ attitudes to four domains of inquiry around which the scholarly discussions about nudging have revolved: consumers’ approval of nudging, consumers’ views on the origin of nudges, consumers’ perceived effectiveness of nudging, and consumers’ concerns about manipulative aspects of nudging.

**Results:**

Interviews revealed that consumers are largely unfamiliar with the concept of nudging altogether. Once defined and explained to them most consumers approve of the concept, especially in the realm of health behavior, given particular conditions: 1. Nudges should be designed for benefiting individuals and society; 2. consumers comprehend the decision-making context and the reasoning behind the promotion of the targeted behavior. Interviews revealed very limited concerns with manipulative aspects of nudges.

**Conclusions:**

These findings call for better information-management to ensure consumers knowledge of nudges and awareness of their current implementation. Under that condition the findings encourage the implementation of nudges benefitting individuals and society in domains that consumers comprehend, such as health behaviors. Further research is required to clarify consumers’ concerns and requirements for nudges in more complex domains such as financial decisions and retirement plans.

## Background

Policy makers in a number of countries have revealed growing interest in novel strategies to improve consumer decision-making. UK prime minister Cameron’s *Behavioral Insights Team* was the first to investigate the possibility of moving from a pure information-driven strategy to improve consumer welfare to behavioral-economics-informed strategies that are no longer based on the image of the purely rational consumer. The United States and Denmark have also recently adopted such innovative approach, while currently both Germany and Belgium are establishing similar groups. These liberal paternalistic strategies, commonly known as *nudges*, influence behavior by changing the way choices are presented in the environment by either presenting them in a more salient or interesting light, or by making them the easier or default option rather than enforcing restrictions or by changing people’s economic incentives [1]. Importantly, nudges promote choices or behaviors that are assumed to benefit the target individual and society as a whole, thereby distinguishing themselves from marketing techniques that primarily benefit the turnover or profit of companies [1,2].

In light of such large-scale interest into the implementation of nudges in combating rising obesity rates, encouraging retirement savings and organ donations, and in improving environmental protection [3], scholars from various academic disciplines have been investigating the appropriateness of nudging as a policy instrument in targeting societal matters. While this multidisciplinary assessment has revealed the high complexity of the question about the appropriateness of nudging, it has nevertheless been deficient of the opinion of the presumably most important group – the consumers themselves, as their concerns and attitudes have remained largely uninvestigated. At the same time though, it remains unclear to what degree consumers have knowledge about ongoing policy interests in employing nudges and about nudges themselves. In response to these missing insights the present article makes a two-fold contribution by employing in-depth semi-structured interviews to investigate UK consumers’ attitudes and concerns about nudging in general, and in dedicating particular attention to the domain of health behavior, an area to which many nudges apply [4]. Consequently, the findings of this study reveal the ideas of the presumably most essential group when examining the appropriateness of nudges, the consumers, which will allow researchers and policy makers to determine when, how, and what nudges are accepted. These findings offer practical implications for researchers and policy makers in the design and implementation nudges.

Throughout the introduction we will first introduce four domains of inquiry, which are based on questions and concerns previously raised by scholars that have provided the foundation for our interviews with consumers. These four domains - 1. *the approval of nudging*, 2. *the origin of nudges,* 3*.the effectiveness of nudging*, and 4. *concerns about manipulative aspects of nudging* - reflect both questions and concerns in previous scholarly investigations and those relevant to the target group of nudges, the consumers. Furthermore, we explain our choice for investigating attitudes towards nudges in the realm of health behaviors specifically.

### Approval of nudging

The concept of nudges is based on *liberal paternalism*, embedded between the more extreme ideologies of liberal markets on the one hand and interventionist states on the other. Nudging is described as libertarian in the sense that people are free to choose what to do, and paternalistic in that people’s choices are guided in the direction of their own, as well as societies’ best interest [5-7] – hence, together, nudges could be qualified as *soft paternalism*. An example that has featured prominently in the previous literature is the promotion of healthy eating in cafeterias. In this example, healthy food is placed more prominently and saliently or is positioned in such way that it is easier to reach compared to less healthy alternatives [8-10]. All choices remain available, while the consumer is nudged towards choosing the healthier food via these choice architectural strategies. Thus, the strategy is liberal as the consumer is not coerced into choosing the healthy food, and it is paternalistic in that the consumers’ behavior is subtly, and often unconsciously, guided towards the *better* options.

The discussion about the appropriateness of nudging is rooted in the debate over the state’s rights and obligations to promote public welfare. While extreme liberals are reluctant to interfere with the natural rights of people, such as property rights, life, and liberty, utilitarian and social contract perspectives, respectively, contend that the state should attempt to maximize societies’ overall welfare, or determine state involvement on the basis of collective decision [11]. Additionally, there is a disagreement over how truly liberal or paternalistic nudges are. Proponents of nudges try to reconcile state intervention with the maintenance of people’s liberties and authority [5] by advocating that interventions are not paternalistic when they do not limit a person’s choices and liberties to behave in any way, especially when there is an option to ‘opt-out’. However, critics argue that although nudges may not restrict the available choices, they limit the possibility to rationally deliberate on the decision-making process of choosing [12]. These opposing positions regarding the issue of state intervention in the promotion of public welfare as well as the definition of liberty drive the dispute on the appropriateness of nudging, as well as the different levels of concern about the paternalistic aspects of nudging. Nonetheless, it is unclear where consumers position themselves in this debate. Therefore, the first objective of the current study is to investigating the consumer perspective on the first domain of inquiry: *Consumers’ approval of nudging* in general and in the domain of health behaviors. Do consumers approve of being influenced despite lacking awareness? Do consumers feel that their choices are limited or that their autonomy is infringed upon? Findings will therefore shed insight to the questions of debate from a consumers’ perspective.

### The origins of nudges

The second factor to present here refers to the problem of which body can define what behaviors and choices should be promoted over others. The demarcation of *good* behaviors and choices is problematic. Essentially, the question revolves around the eligibility for the right to declare specific behaviors and choices as *good or better* compared to others. For critics liberal paternalistic policies are based on social norms, shared realities, and familiarity that define particular behaviors as superior to others [3]. For instance, current societal and medical discourses describe healthy lifestyles as superior to unhealthy lifestyles, where they consider long, healthy lives as the ultimate goal, slim and fit bodies as the indicators of a healthy lifestyle, and all the while promoting behaviors to align with these norms. Such discourse is persistent despite the lack of consistent support for the notion that slimness is a major factor contributing to long-term health [13]. In promoting these aligned behaviors policy makers reinforce the existing social norms and shared realities [14,13], thereby promoting the health of some members of society while simultaneous leading to increased stigmatization of those members not willing or capable of behaving in accordance with these prescribed norms [15]. In light of these arguments, this study explores the second domain of inquiry: *Consumers’ opinions regarding the origin of nudges*. In other words, do consumers care or have concerns over who designs the nudges? Are consumers concerned about the definition of *good* behaviors?

### The effectiveness of nudging

A factor of more practical relevance refers to the effectiveness of nudging in changing long-term behaviors and value structures. Critics of nudging question whether the design of choice architectures leads to long-term changes in people’s behaviors and value structures [7]. They claim that substantial behavioral impact leading to long-term healthy or sustainable behaviors requires consumers’ recognition of the urgency to change lifestyles and subsequent conscious behavioral adjustments. Merely being nudged into these behaviors without deliberation is judged as an insufficient, short-term strategy [7]. Furthermore, marketers can easily counteract uninformed behaviors caused by nudges in an attempt to increase sales and maximize profit. Consequently, these opposing forces could lead to a system in which large amounts of public finances are invested into nudging behaviors that benefit society and consumers which are simultaneously neutralized by marketing strategies guiding choices and behaviors in the opposite direction [7,15]. This aspect is investigated in the current study by examining the third domain of inquiry: *Consumers’ perceived effectiveness of nudging.* While this perception does by no means translate into an objective evaluation of the effectiveness of nudging, it contributes to an understanding of consumers’ attitudes toward the usefulness of nudges.

### Concerns over the manipulative aspects of nudging

A final point of concern is the potentially manipulative nature of nudging. This factor of concern is essentially an extension of the considerations raised in the first domain of inquiry, the approval of nudging. As mentioned in that first paragraph, opponents of nudges critique the paternalistic aspect of nudging, the idea that nudging may potentially limit the possibility for consumers to rationally deliberate on the decision-making process by promoting particular choices outside their conscious awareness [6,7,12]. Accordingly, the fourth domain of inquiry explores consumers’ opinion on this aspect and whether they have *concerns about the manipulative aspects of nudging*, as raised by the critics.

### The case of health behaviors

Health behaviors are prominent targets of recently implemented nudges that have been subject of scientific investigation. These nudges specifically target behaviors such as smoking, dieting, physical exercise, and alcohol consumption [16]. Health behaviors are a good candidate for developing nudging interventions for two main reasons: Firstly, most members of society want to lead healthy lifestyles and at the same time report problems in adhering to this goal, especially in light of short-term temptations. These problems can be the result of health-illiteracy or limited self-regulatory skills, which explains the ineffectiveness of information-based approaches to promoting healthy lifestyles [17]. These factors imply that the promotion of health behaviors is particularly suitable to nudging [18]. Secondly, health behaviors are often driven by habits and impulses and are therefore little subject to rational considerations [4]. As such, health behaviors align particularly well with the functioning of nudging in the sense that they avoid conscious deliberations about choices and instead promote behaviors via relatively unconscious routes, making healthy behaviors easier and healthy choices more salient [1].

There is good reason for policy makers to be concerned with the promotion of health behaviors considering the increasing number of people with obesity, and especially the increase in overweight children, as well as consequent health problems such as diabetes, cardiovascular diseases, and cancers [16]. Despite this growing interest in nudging strategies, governments and policy makers are concerned with the acceptability of such interventions by the public, due to the concerns raised in the scholarly debates described above. In response to this, researchers have been calling for investigations into consumers’ acceptability of nudges and concerns about being nudged [4,18]. A first investigation by Diepeveen and colleagues [16] examined electronic databases and empirical studies reporting attitudes towards health interventions, including nudging strategies in health behaviors. This investigation revealed strongest acceptability of strategies targeting others rather than the self and less intrusive strategies. Yet, this study did not directly assess consumers’ attitudes and concerns related to nudging as is required for a holistic, in-depth understanding of consumers’ reasoning. This gap of knowledge is to be filled by the current study.

### Research question

The aim of this research project was to examine consumers’ knowledge of and attitudes about nudging in general and nudging in a health domain as well as their concerns about being nudged. To obtain an understanding of consumers’ attitudes and concerns about the aspects of nudging that feature prominently in the scholarly discussions this projects investigated four domains of inquiries, each of which relates to one point of discussion among scholars mentioned previously in the introduction. As such we investigated (1) *consumers’ approval of nudging* by uncovering consumers’ familiarity with nudging, their attitudes towards nudging in general and nudging within a health domain; (2) consumers’ views on the *origin of nudges* by exploring their attitudes in regards to who designs nudges and determines behaviors to be promoted, (3) consumers’ perception in how they judge *the effectiveness of nudging*, and (4) and consumers’ concerns with nudging, and potential *manipulative aspects*, as a strategy of improving consumers’ behaviors. As no explicit hypotheses about these attitudes and concerns were specified, the research was essentially exploratory in nature and targeted at examining any associations consumers had in relation to nudging.

## Methods

### Semi-structured interviews

In addressing these research questions a qualitative, exploratory design was implemented. The researchers held semi-structured in-depth interviews with consumers in an informal communication setting in order to obtain as many ideas, associations, attitudes, and concerns people may have in relation to nudging [19]. The semi-structured interviewing method was chosen because it allows for both structure and flexibility. The structure of semi-structured interviews allows interviewees to answer questions as set out in an interview guideline addressing the research questions under examination, with their responses fully probed and explored. Meanwhile, the flexibility of semi-structured interviews allows the researcher to be responsive to the relevant issues raised spontaneously by the interviewee [20]. As such, while the interview guideline provided basic questions to be addressed in specific phases of the interview, questions varied between interviews as a natural progression of the situation as well as the input from interviewees.

The interview guideline specified four phases to provide a structured framework addressing the domains of inquiry presented in the introduction. In *phase 1*, interviewees were prompted to explain their familiarity with nudging and their general attitudes without the provision of a clear definition for nudging. For example interviewees were asked whether they had ever heard of the concept of nudging and whether they could explain what they understood it to be. In *phase 2* the same questions were asked in reference to nudging in a health behavior domain. In *phase 3* the interviewer provided a definition of nudging which included two main aspects. Firstly, nudges were defined as subtle cues designed to help people make better choices and behave more optimally which may or may not occur outside of conscious awareness. Secondly, nudges were defined as influences on behavior by the way choices are presented rather than by removing choices. To facilitate understanding of the concept examples of nudges were provided including the distancing of color printers to prevent unnecessary use of color prints; the use of colored bin bags to ease the separation of waste; and the provision of smaller plates in a cafeteria to prevent eating large portions. Based on this definition and the examples interviewees’ general attitudes and concerns were collected. For example, interviewees were asked what they thought of these nudges, whether they would appreciate being nudged, and whether it mattered to them who designed these nudges. Additionally, attitudes and concerns relating to nudging in the health domain were targeted by providing more examples of health-related nudges such as exchanging unhealthy snacks at the cashier with healthier snacks; placing healthy snacks more prominently on shelves in supermarkets; and downsizing the serving plates at all-you-can eat buffets. In *phase 4*, questions were presented about the acceptance of nudges targeted at the interviewee him/herself. Specifically, interviewees were asked whether they would approve of being targets of nudges, whether there are specific domains in which they do/do not accept behavioral guidance, and whether they believe in the effectiveness of nudges on their own behavior.

### Participants and procedure

To ensure access to the attitudes and concerns of a broad range of societal groups, a sample of participants was recruited through a marketing research company that represented a large variety in terms of age, socioeconomic status/educational background, gender, and BMI of the participants. It was anticipated that having interviewees with varying backgrounds in terms of age, socioeconomic status/educational background, and gender would improve the representativeness of the sample. Socioeconomic status (SES) and educational background were accounted for on the basis of the UK demographic classification scheme (National Readership Survey social grades) which classifies citizens as high SES A and B (N = 5), middle SES C1 and C2 (N = 8), and low SES D and E (N = 7). Furthermore, as a particular focus of the current study relates to healthy eating behavior, we included interviewees with varying BMI scores (i.e., normal weight, overweight, obese. Interviewees were matched on their BMI classifying underweight < 18.5 (N = 1), normal weight 18.5 – 24.9 (N = 8), overweight 25–29.9 (N = 10), and obese > 30 (N = 1) interviewees. All interviewees were recruited from public settings in London and invited to participate in interviews for monetary reward. The resulting sample consisted of 21 interviewees of whom one was excluded due to limited English proficiency.

Prior to each interviewing session, all participants were informed about the nature of the semi-structured interview. It was explained to participants that they would be asked to discuss and express their opinions on a specific topic, and they would not be obligated to respond should they feel uncomfortable at any stage of the interview. Furthermore, participants were informed that the interviews would be recorded for research purposes (i.e., data analysis at a subsequent stage), and it was emphasized that the contents of interviews would be kept anonymous at all times. It was made known to the participants that there would be a possibility that direct quotes would be presented in a published research report, but that their anonymity would be ensured. The interviewing session began after participants have provided verbal consent for the interview to be recorded. The interviews lasted for a maximum of approximately 40 minutes. At the end of the interview, each participant were provided with an opportunity to ask questions, thanked and compensated with monetary reward for their participation. This study was conducted in accordance with the ethical standards described by the Medical Research Involving Human Subjects Act [21], which exempts research on healthy human subjects from review for as long as it does not involve any invasion of participants’ integrity. Consequently, no formal ethical approval was required according to Dutch national standards. Nevertheless, ethical approval was obtained at Utrecht University for the EU funded FP7 umbrella project Marie Curie Fellowship Consumer Competence Research Training (CONCORT), a European network collaborating the research efforts of 14 Early Stage Researchers from various academic disciplines dedicated to generate research improving consumer welfare. The current study is part of the research effort directed under CONCORT. Furthermore, the UK market research agency operates under and is member of the Market Research Society Code of Conduct.

### Analysis

#### Thematic analysis

All recorded interviews were first transcribed and subsequently subjected to thematic analysis. The thematic analysis aimed at finding key patterns of ideas and attitudes in the interviews by coding for recurring codes and themes. Throughout the process coders were interested in those responses by interviewees that related to the research questions. A semantic approach was employed that focused on a description of the interviewees’ responses rather than the interpretation of these responses [22].

The analysis was based on Braun and Clarke’s [22] step-wise procedure. Two coders (the same as interviewers) familiarized themselves with the interviews and transcriptions in the first phase of the analysis. During this phase, using a deductive approach, the coders independently collected preliminary codes that identified extracts of data containing meaningful information relevant to the research questions. These preliminary codes were subsequently compared, discussed, and revised by the two coders. In a subsequent step, codes were connected together based on repeated co-occurrences (i.e., they were frequently detected in natural clusters in the transcriptions) and semantic relationships (i.e., they depicted a concept when manually put into proximity) into overarching themes [23]. No numeric requirements were set for determining the existence of a theme or code but their occurrence and prominence determined the classification. These overarching themes were named in a manner that described and interpreted an aspect of the data that was relevant to the research questions. This process led to the final coding scheme including both themes and codes accompanied by a definition and an example (see Table 1). Afterwards, a second round of coding was performed where the established codes from the final coding scheme were independently applied to the transcribed interviews. In cases where codes diverged between coders explanations and discussion led to an agreement in all cases.

**Table 1:** **Coding scheme**

**Theme**	**Code**	**Definition & example**
Knowledge	Familiarity	Acquaintance with the concept of nudging.
		E.g., [“The topic that I would like to talk about is nudging. Have you ever heard of nudging?”] “I have never heard of it.”
	Observed Nudges	Examples of nudges.
		E.g., “There are these signs, neon signs, an electronic sign that shows you a sad face when you’re going above the speed limit or a nice smiley if you’re ok.”
	Novel ideas	Suggestions for domains for new nudges.
		E.g., “I think walking more around London is a good way. I know they encouraged more cycling but I think people should walk more.”
Individual differences	Objective	Differences in peoples’ motives.
		E.g., “I think of people are willing to do the right thing and willing to be healthy, I think…..”
	Indifference	Lack of interest in target behavior.
		E.g., “There are a lot of people who care about it but you get certain people who don’t. They just do it because they just can’t be bothered to put it into the other bags.”
Self-target	Approval	Level of agreement with being nudged for the self.
		E.g., [“Would you appreciate it if you were nudged into healthy eating?”]
		“Yes. I would appreciate it. I think everyone wants to do it and it is great to be encouraged to do it.”
	Effectiveness	Judgment of the extent that nudging would be successful when targeted at the interviewee.
		E.g., “Personally I don’t think I need any nudges but I guess it helps, yes. I am generally quite healthy anyway.”
General - target	General Approval	Level of agreement with nudging targeted at anyone
		E.g., “I think the food is an absolutely brilliant idea, absolutely brilliant because we have got so much obesity and it is too easy for them to go and grab a big plate, fill it up and then just go back again but if you have got something smaller then you can only eat what is on the plate if you like and I think that is a good thing. I think that would help a lot of people. The stairs is good too because it makes it fun because sometimes exercise can be so boring.”
	General Effectiveness	Judgment of the extent that nudging would be successful when targeted at anyone.
		E.g., “No, what I am saying is, it has its benefits so people who alright yeah, who go to the supermarket and take the back and read it looking at the calories because they are health-conscious but for those that are not they can see a healthy food and just pass it back. So being there means nothing to somebody who has no idea.”
	Specific target groups	Potential population segments targeted by nudges.
		E.g., “So yeah, I think it would be important and from a children’s perspective as well because in supermarkets sweets are deliberately put by the checkout in order for a child to spot them and also last minute shopping so it is all psychological.”
Origin	Actors	Individuals or groups implementing or designing nudges.
		E.g., [“Would it matter for you who is deciding on what is a good behaviour?”] “Probably the dieticians or the doctors.”
	Expertise	Required level of knowledge in the targeted behavior.
		E.g., “Someone who, maybe a nutritionist or something like that because they obviously knows about health things or someone who has done psychology as well and knows why people are going to pick things. Perhaps a psychologist and a nutritionist.”
	Intention	Motives of the agents involved in designing nudges.
		E.g., [“Does it matter who implements these health nudges or who decides on what the good behaviour is?”] “It doesn't matter as long as the goal is clear that it is to help people lead healthier lives.”
	Trust	Degree of confidence in agents’ motives related to the design of nudges.
		E.g., “I would trust somebody that had done their research and it is maybe Government funded or maybe a Government initiative or a health initiative so something that has got a sort of, a reputable backing.”
Behavior	Habit	People’s routine behaviors.
		E.g., “In retrospect, the nudges then hopefully become part and parcel of your life and your everyday working life or home life.”
	Individual Capacity	People’s extend of influence on their own behavior.
		E.g., “Yes actually yes, because we try to push ourselves but sometimes something else influences it, you know what yeah I am going to do it.”
	Facilitation	Supportive effects of nudges on behavior.
		E.g., “As long as people have opinions but make it easier for them to choose the more healthier option.”
	Social environment	The relationship between people’s behavior and their social surrounding.
		E.g., “It might change you one day to say “Come on, everybody is so I might as well” and it is good for the future.”
Freedom of choice	Coercion	Oppressive influences of nudges on behavior.
		E.g., “What you do is you manipulate their decision making whereby it is them noticing that you are doing it or them not noticing that you are doing it, it doesn’t matter. You just manipulate them to do what you want them to do.”
	Nudging-suitable domains	Appropriateness of behavioral domains for nudging.
		E.g., “I don’t know how you can nudge in those areas because there is so much out there, there’s so much and it is personal choice isn’t it? It is personal belief in terms of religion.”
	Choice-set limitation	Restricting the availability of choices and possible behaviors.
		E.g., “I think alternative options are always good like if you had an alternative option but I don’t think they should take anything that is currently there and then say you can’t have that any more.”
Cognition	Reactance	Counter-reaction to the promoted behavior.
		E.g., “There are people who are set in their ways and bringing in anything that is going to be far from their norm, even if it is a simple task, is not going to go down well with them and there are those people who just don’t like change. Even if you bring it, you might want to resist.”
	Awareness	(No) Realization of the influence of nudges.
		E.g., “I think we are nudged every day in life and we don’t realise we are being nudged.”
	Need for information	Required level of information on being nudged and/or the targeted behaviors.
		E.g., “Because they are trying to encourage healthy eating and it is educating people because information is power. If you know the good and the bad things, I hope there are going to be loads of advertisements about these things because people need to be educated and they need to be aware of things before they can be applied in practice.”

## Results

The results are structured according to the four domains of inquiry based on information extracted from the interviews using deductive coding, for an overview of the codes and resulting themes that were used to identify relevant information pertaining to the research questions, see Table 1. We would like to emphasize that the goal of this investigation was to learn about any representations, thoughts, attitudes, and concerns consumer may have on the matter of nudging rather than to provide a numerical overview of the distribution of these opinions. Citations provide examples of responses from interviewees but are selected for demonstration purposes rather than representativeness.

### Consumers’ approval of nudging

This first domain of inquiry uncovered consumers’ familiarity with nudging, their attitudes towards nudging in general and nudging within a health domain. Despite the vivid discussion around nudging in the scientific community as well as frequent coverage on media outlets, interviewees were largely unfamiliar with the concept of nudging as influences on behavior. If interviewees voiced any interpretation of what nudges could be understood it in its literal sense of poking or (gentle) shoving.

*“In a poking kind of sense or some applications to send someone a nudge”*

(Male, 27, high SES, overweight)

Due to this general unfamiliarity most interviews moved directly into phase 3 of the interview guideline in which interviewees were introduced to nudging via the provision of a definition and examples from first the general nudging domain and later the health-related nudging domain. While some interviewees could relate to these examples, i.e. reported having observed similar nudges, it did not remind them of having heard of the concept of nudging as influence on behavior prior to the interview. Nevertheless, some interviewees reported being familiar with the concept of the subtle, unconscious influences, however, more in the context of marketing techniques that surround people in everyday life.

*“Advertising in a sense is a nudge about a product”*

(Male, 29, middle SES, normal weight)

During the interview a distinction was made between approval of nudges in general, approval of nudges in the domain of health behaviors, nudges applying to people in general as well as those applying specifically to the interviewee. Additionally, interviewees were asked whether there were any domains in which they would consider nudging inappropriate.

In principle, interviewees reported to appreciate the idea of nudging as a whole without seeing negative aspects.

*“No. I don’t think there is a disadvantage because at the end of the day it is to create a safer and a better environment. If they don’t agree with it then I guess they just don’t have to do it if they don’t want to but at the end of the day it is a benefit for everyone”*

(Female, 28, middle SES, normal weight)

While the initial responses were mostly positive, some interviewees also reported these nudges to be related to manipulations. Nevertheless, throughout the interviews a strong majority appreciated nudging as a whole and even more so when they target health behaviors. Interviewees could relate to the difficulties revolving around health behaviors on a societal level as well as related to their own health behaviors.

*“I am all for it. Anything to do with health behaviour and improving people’s health in general, I am always supporting that. I think it is a very clever idea because no one likes change because if you tell people “Do this” then they will do that. There won’t be a good reaction. But I think nudging is in some ways subconsciously trying to get people to do or to make a better choice, so yeah I support it”*

(Male, 27, high SES, overweight)

While interviewees differed in the degree to which they consider health-related nudges applicable and necessary for themselves this did not reduce their support. Even in cases where interviewees considered health-related nudges unnecessary for themselves they remained supportive of nudges targeting society as a whole including themselves.

*“Yes. I would be more in favour. I think it’s needless for me. In the country everyone is getting fatter so the teenagers coming into these buffets, if they were having smaller plates and they had smaller plates at home they wouldn’t think “I will eat more”. It might help”*

(Male, 24, high SES, normal weight)

Considerations of manipulations when investigating attitudes to health-related nudges specifically remained very rare.

Approval of nudges appeared to be related to the intentions of the nudging body/institution. The positive attitudes towards nudges were driven strongly by the idea that nudges are designed with the intention of improving peoples’ behaviors for the better of society and themselves. This requirement was often mentioned as the basis for approval and became most evident in the case of health-related nudges were good intentions were understood as helping people behaving in a more health-promoting way. For nudges in the general domain, interviewees were particularly appreciative of nudges relating to environmentally friendly behaviors such as separating waste and keeping streets clean.

*“Like I said before, anything that promotes good behaviour and living healthily is part of good behaviour, I think it’s good, it is a good idea”*

(Male, 48, low SES, overweight)*“*

Disagreements with the concept of nudging as a whole or in relation to health-behaviors were not encountered during the interviews. Nevertheless, some interviewees raised concerns, mostly upon probing for negative aspects of nudges, that nudges and behavioral influences were similar to manipulations, which raised concerns with the concept. However, interestingly, these concerns were described as manipulations common to standard marketing practices, such as placing products in shelves to increase attention to particular choices. These considerations will be further discussed in the results on *concerns about manipulative aspects of nudging*.

*“It depends on what kind of thing it was, I suppose and what kind of decision it was that they were trying to force you into. If it was an environmentally good thing then I wouldn’t mind if someone is making these nudges but if it was something to do with making me pay out for something that I don’t necessarily need and they are just trying to force it upon me then I would find that negative”*

(Male, 24, high SES, normal weight)

Whereas interviewees had difficulty reporting any behavioral domains for which they would not appreciate nudges, with few exceptions mentioning financial domains, they did raise concerns regarding nudges targeted at particular groups such as children, while in other examples children are considered a particularly good target group. Based on the argument that children are easily manipulated nudges targeting children were rejected by some of the respondents. This rejection was irrespective of the fact that nudges were defined as based on good intentions and with behaviors improving outcomes for the target population in mind. There were both expressed support and concern over the exposure of nudging to children:

*“So yeah, I think it would be important and from a children’s perspective as well because in supermarkets sweets are deliberately put by the checkout in order for a child to spot them and also last minute shopping so it is all psychological”*

(Female, 59, low SES, normal weight)

*“With children maybe and maybe that is too pushy in that sense because it is not being explained. It is just being forced on them if you like. Yeah, maybe in children but not in adults, no. I think it is fine”*

(Female, 46, low SES, overweight)

### The origin of nudges

Interviewees generally expressed that if the intention behind the nudge was good, as most agreed in the case of health behavior and healthy eating, they would not be particularly concerned with the actors who design or implement the nudges. Furthermore, interviewees also mentioned that because they would not be immediately aware of the presence of the nudge due to its subtle nature, the actor hence becomes irrelevant for them to consider. Nonetheless, some interviewees suggested that if the nudges were targeted particularly at healthy eating, they would have greater trust in actors who have a reputable backing and specialized expertise in the subject. For example, in the domain of health and food, some interviewees expressed their trust in doctors, dieticians, or nutritionists. Psychologists were also considered as good candidates for designing nudges as they would have knowledge of consumer behavior and the factors that shape people’s choices. To illustrate, when discussing potential actors for nudges for healthy eating, one respondent said,

*“Someone who, maybe a nutritionist or something like that because they obviously know about health things or someone who has done psychology as well and knows why people are going to pick things. Perhaps a psychologist and a nutritionist”* (Male, 29, middle SES, normal weight)

Trust in governments or politicians was mixed. On one hand, the Government was spoken about as an actor who has the authority and the responsibility to guard and improve the welfare of its citizens, and therefore should exercise its influence by directing health behavior initiatives though the implementation and design of nudges. On the other hand, as one respondent quoted,

*“…anything Government-related or anything that comes from the Government people instantly distrust. Because the Government is coming from a discredited stance a lot of times to start with. So based on that people are not going to take what they say. They said about the meat that people were eating and how it was the Government knew that was all this type of meat that we were being served and they said – Let them still eat it – and stuff like that”*

(Male, 56, high SES, overweight)

Interviewees also voiced that they would not appreciate being nudged into behaviors or choices by actors such as marketers with commercial purposes of gaining profits for a company. Nonetheless interviewees recognized that this is inevitable, and is in fact quite an existing mundane scenario in everyday situations.

*“I mean it is all about marketing in this particular case. And since here is always going to be somebody trying to, I guess the word is manipulate other people so it might as well to be somebody who has, thinks of ways to help them and somebody else that might think a bit more about the money and not so much about what is good for people”*

(Male, 34, middle SES, overweight)

This quote described a general sense of consensus amongst interviewees in approving actors in designing and carrying out nudges, given that they are dedicated to promoting the wellbeing of consumers and the general public, as counter efforts to companies and marketers whose aim is to increase commercial profits and private gains.

### Consumers’ the perceived effectiveness of nudging

Nudging was overall approved by interviewees, but as a general concept it was too abstract for interviewees to judge its potential/expected effectiveness. However, given some examples interviewees discussed the effectiveness of nudges more fluently. According to interviewees, what made nudges potentially effective was that they subtly facilitated the targeted behaviors. Similarly for health behaviors and healthy food choices, nudges were regarded effective because they made healthy behaviors easier or more fun to perform, and made healthy food choices more salient. As such, the nudged behavior became easy to adopt and to carry out as a habit, and eventually be integrated into the social environment that further endorses the behavior. Furthermore, targets’ individual objectives and capacity to influence their own behavior were considered as important contributing factors. Interviewees acknowledged that considering the recent focus on issues surrounding food, health, and obesity in the media and public discourse, most people generally have an awareness of behaving healthily, although the level of intention varies between individuals. As such, nudging was rated as effective for those who already have an intention to eat healthily and are taking actions to fulfill this goal.

On the other hand, interviewees who, in their opinion, already have a successful individual capacity for healthy behaviors evaluated nudges to be less effective when applied on themselves, but nonetheless would appreciate the potential benefits.

*“Personally I don’t think I need any nudges but I guess it helps yes. I am generally quite healthy anyway”*

(Male, 29, middle SES, normal weight)

Overall interviewees considered nudges to be effective for the society as a general target, and in most cases for themselves as targets. Nonetheless, nudges were not considered useful for individuals who have no intention or are indifferent to healthy eating.

*“Someone that really doesn’t care, it is going to be quite hard to nudge them”* (Female, 28, middle SES, normal weight)

Furthermore, price was considered as a significant determinant in people’s food choices. As such, some interviewees saw price as a potential obstacle to the effectiveness of nudges in promoting healthy food choices, considering that some people choose the cheaper option regardless of the food product’s nutritional value.

Finally, the need for information was mentioned as a factor that could contribute to the effectiveness of nudges. Although nudges were intended to be subtle and not explicitly instructive, interviewees felt that people would need to have an initial understanding of the importance of health behaviors before they could benefit from a nudge. For example, it was suggested that complimentary information such as the benefits of healthy eating could be presented adjacent to the nudge in order to increase its effectiveness.

### Concerns about manipulative aspects of nudging

When examining consumers’ concerns as to the manipulative aspects of nudges a minority of interviewees showed concerns over the freedom of choice offered by nudges. The main hesitation was that the interviewees would potentially lose autonomy over their decisions or that there would be a limitation to their choice set.

*“There will be a problem if you are saying people shouldn’t eat junk food or if you take away the elevator”*

(Female, 30, high SES, overweight)

When discussing nudges without a particular context, only a few interviewees demonstrated skepticism and hesitation, as they understood the influences of nudges and manipulations as employed but actors such as marketers in a similar light. Interviewees also expressed that they would not appreciate if they realized that they had been led to a decision that was out of their awareness. This did not necessarily mean that they did not want to be nudged, but if so, they did not want to detect the influence.

*“But the disadvantage of it is if it is something negative and if the customer of the person finds out that things are actually strategically placed or done for that reason and they might be offended”*

(Female, 27, middle SES, overweight)

Nonetheless, this feeling of coercion was mainly limited to nudges intended for marketing purposes, or that the intention behind the nudge was not to the best of their interests. Considering that, by definition, these influences are not nudges, they should not be understood as resistance to appropriately implemented nudges but to other external influences on behavior.

*“but if it was something to do with making me pay out for something that I don’t necessarily need and they are just trying to force it upon me then I would find that negative”*

(Male, 24, high SES, normal weight)

*“Although it was the right thing that I had got but I had been maneuvered there. Some people would rather take the wrong thing but it was their choice”*

(Male, 56, high SES, overweight)

On the other hand, nudging in the domain of health behavior, there were no concerns about coercion from the part of the interviewees. Particular to healthy eating, the general perspective was that nudging was more of a facilitation of better choices rather than a manipulation of choices. Interviewees also indicated that there were clear benefits to healthy eating; therefore they would not be concerned if they were nudged into healthier choices out of their awareness.

Additionally, interviewees implied domains such as religion, politics, and contraception would not be suitable nudging domains as they involved individuals’ expression of personal beliefs.

*“For example in schools now, I am of Christian and I have been brought up to understand that marriage is between a man and a woman. I am being told, I have come to know that there are silent nudges that try to force same-sex marriage or same-sex down the throats of people at churches […] no matter what orientation you choose but they are slowly taking away that freedom. How do I explain it, sometimes nudging feels like a propaganda by certain people in the Government to force”*

(Female, 30, high SES, overweight)

## Discussion

The main conclusion of the interviews is that consumers are generally appreciative of nudging both as a general concept and when targeting health behaviors. While a surprisingly high unfamiliarity with the concepts was revealed this unfamiliarity further justifies the study’s rationale in involving consumers in the discussion over the appropriateness of nudging and the implementation of nudges. At the same time it raises the question of whether consumers are sufficiently familiar with nudging strategies to provide sophisticated and elaborate attitudes toward the concept. Considering the lacking familiarity with nudging prior to the interviews consumers may have provided a rather crude attitude toward a concept defined and explained to them. This issue by no means implies that consumers should not be involved in judging the appropriateness of nudging. It does, however, indicate a need for increased consumer information about these already ongoing strategies and stronger consumer involvement in determining their appropriateness. Thus, the findings yield the question: Who should judge a nudge? And are policy makers sufficiently informing and involving the target group of nudging to ensure Thaler and Sunstein’s [1] requirement of transparency and the possibility to opt out?

Looking into the general attitude towards nudging most eloquent approvals were encountered when communicating about examples of nudges, which may have aided interviewees’ understanding of the concept as well as the reasons for the promotion of particular behaviors. Employing examples, especially examples of health behavior, helped demonstrating the difference between a good behavior that should be promoted and a bad behavior that should be avoided. As such, it may be the case that nudging receives particular support when consumers understand the reasons for promoting, as is the case for health behavior, but lower support when it is discussed in general, abstract terms, which are more complex to grasp. Despite the general approval interviewees were hesitant in forming an opinion regarding the appropriateness of nudging in areas such as religion and politics, as these domains were subjective to personal beliefs and moral value.

Good intention behind nudges was the main driver for approval of the concept. When interviewees reported negative aspects they mostly referred to restricting choices (which by definition is not part of nudging) or a disapproval with being influenced in principle. On the other hand, standard marketing techniques were sometimes compared to nudges, but people readily distinguished marketing as a source of negative external influence, because unlike nudges, the targeted behaviors by marketing techniques were not always in the interests or advantage of the consumers. At the same time, consumers did not question how and why a promoted behavior would be considered a *good behavior.* Yet, it remains unclear to this point whether this lacking scrutiny derives from a strong trust in the sources of nudging, a general disinterest, or an uncritical acceptance of the existing discourses about health behaviors.

While there was no clear preference for who should design or implement nudges, this was only under the general assumption that the origin of nudges endorsed good intentions. Interviewees generally perceived an intention to be *good* if it pursued a clear objective in promoting positive behaviors for individuals and society. Given this circumstance, nudging for the promotion of health behaviors was widely approved considering that there are clear distinguishable benefits and negative consequences associated with health. Related to this was the notion of freedom of choice. A minority of interviewees voiced concerns over the potential choice limitations or coercive directions imposed by nudges. However, these concerns were not weighted as heavily given that nudges ought to be based on good intentions to benefit the recipients or the greater society, such as the case for promoting healthy or environmentally friendly behaviors.

Finally, there was awareness that while nudging could be implemented to promote positive behaviors amongst the masses, its effectiveness was sensitive to individual differences of the recipients. Specifically in the context of health behavior, nudging was judged to be less effective for those who already have a good personal capacity and are successful in managing and conducting these behaviors. For example, interviewees who, in their opinion, already have a successful individual capacity for healthy behaviors evaluated nudges to be less effective when applied on themselves, but nonetheless would appreciate the potential benefits. Furthermore, a disregard or indifference to the value of health was suggested to potentially undermine the influence of nudges toward health behavior or choices.

Nonetheless, the outlook on nudges was that they would be an effective strategy because they are subtle and could be easily integrated in the everyday environment; and since the general public has a fundamental understanding of the advantages and values of good health, most people could benefit from the facilitation of nudges in performing healthy behaviors.

In light of the ongoing current debate surrounding the ethics and implementations of nudges in the academic and political arena, there is a dearth of research investigating the perspectives of consumers, who are the ultimate targets of nudging. Responding to the call for research investigating acceptability of nudges and concerns over being nudged [4,18], the current research is the first to our knowledge to examine this topic by directly reaching out to consumers. While the findings of the current study shed light into a less-explored research territory, it contains certain limitations. First, the interview questions included in the semi-structured interview schedule were strictly linked to the current research’s overarching research questions. This choice could have potentially limited the findings that may have emerged if the interviews were open-ended and fully participant directed. Similarly, only deductive coding was employed in order to extract data from the interviews that were directly relevant in answering the main research questions, which may have prevented interesting, but less research topic-relevant findings to surface. Another inherent limitation of qualitatively interviewing is that interviewees’ responses are subjected to social desirability and demand-characteristic effects of the interview situation [24]. Finally, as our findings revealed the extent to which consumers were familiar with the concept of nudging was minimal, this raises the question as to how much and how accurately consumers would be able to convey their attitudes and perspectives on a concept that they do not have substantial knowledge in. The issues mentioned above may have influenced the validity of the data, but the findings of the current research serve as a first starting point to examine consumers’ attitudes and concerns about nudging and to stimulate future research using more rigorous scientific methods in examining a topic that requires much research attention.

## Conclusions

These revelations are particularly important in light of the current scholarly discussion as to the appropriateness of nudging. While this discussion is relevant and theoretically interesting, it should not function as a basis for deciding for or against the implementation of nudges. In contrast, the attitudes, concerns, and requirements of the target group – the consumers - should be considered as an additional source of such decision-making. At the same time, this study uncovered a lacking familiarity with the concept of nudging and possibly insufficiently critical reflections of these strategies on the side of the consumers. Considering the moral need of including consumers into the process of judging nudges this finding calls for improved consumer information about nudging strategies and stronger consumer involvement into judging their appropriateness to ensure safeguarding mechanisms such as the possibility to opt out of unappreciated influences on behavior.

Meanwhile, in contrast to the scientific debate, we find no direct justification to reject nudging, especially within the realm of health behavior for which consumers understand the benefits of promoted behaviors nudging strategies. However, these conclusions cannot be conclusively drawn for other behavioral domains. Additional research will be required to determine consumers’ acceptance and concerns with nudges in the domains such as financial decision making, fund raising, organ donations, and many more. Furthermore, due to the qualitative nature of the study no deliberate procedures were taken to obtain quantitative data. Our findings suggest a majority of approval for nudges but there is no precise quantitative information about the distributions of these opinions. Future research is encouraged to employ quantitative measures to explore and measure the distribution of public opinion on nudging in order to compliment the current findings.

For governments currently employing or considering the implementation of nudges and paternalistic strategies into their range of policy instruments the findings speak in favor of such strategies despite criticisms from some scholars and media while simultaneously call for more information about nudges. However, the findings shows that nudges are particularly accepted in behavioral domains consumers comprehend. Consequently, information-based approaches and nudging strategies should go hand in hand to achieve both acceptance of the strategies and improvements of consumer welfare. Nudges should neither be rejected on the basis of philosophical concerns, nor be implemented blindly, without providing information to the consumer as requested by proponents of traditional information-based approaches.

## Abbreviations

SES: Socio-economic status
BMI: Body Mass Index
